# Association of Adult Depression With Educational Attainment, Aspirations, and Expectations

**DOI:** 10.5888/pcd17.200098

**Published:** 2020-08-27

**Authors:** Alison K. Cohen, Juliet Nussbaum, Miranda L. Ritterman Weintraub, Chloe R. Nichols, Irene H. Yen

**Affiliations:** 1 Department of Public and Nonprofit Administration, School of Management, University of San Francisco, San Francisco, California; 2 School of Public Health, University of California, Berkeley, Berkeley, California; 3 Graduate Medical Education, Kaiser Permanente, Oakland, California; 4 Department of Public Health, School of Social Sciences, Humanities and Arts, University of California, Merced, Merced, California

## Abstract

**Introduction:**

Social factors across one’s lifespan may contribute to the relationship between low educational attainment and depression, but this relationship has been understudied. Previous studies assessing the association between educational attainment and depression did not fully account for prior common determinants across the life course and possible interactions by sex or race/ethnicity. It is also unclear whether the link between educational attainment and depression is independent of the role of aspired educational attainment or expected educational attainment.

**Methods:**

We used generalized linear log link models to examine the association between educational attainment at age 25 and depression at age 40 in the National Longitudinal Survey of Youth 1979 cohort, adjusting for confounders and mediators from childhood, adolescence, and adulthood.

**Results:**

Members of each educational attainment group were less likely to be depressed at age 40 than those with less education. After adjusting for educational aspirations and educational expectations, the risk ratios became closer to the null. Neither sex nor race/ethnicity interacted with educational attainment. Additionally, low educational expectations in adolescence, but not low educational aspirations, was associated with a higher risk of depression at age 40.

**Conclusion:**

Our study provides a nuanced understanding of the role of education, educational expectations, and educational aspirations as part of education’s effect on risk of depression after controlling for a thorough set of confounders and mediators. Our findings may help advance the study of social determinants of depression.

**Summary**

**What is already known on this topic?**

Educational attainment is associated with depressive symptoms and may affect depression through various socioeconomic pathways. Education may offer opportunities for developing interventions to reduce the disease burden of depression.

**What is added by this report?**

We examined key factors in early childhood and adolescence often omitted as confounders of the relationship between adolescent educational aspirations and expectations and mental health outcomes in adulthood. We also looked at differences by sex and race/ethnicity.

**What are the implications for public health practice?**

Our findings suggest that the social forces that constrain education may also affect health. Additionally, our findings support other research that encourages health and education practitioners to acknowledge educational interventions as public health interventions.

## Introduction

Depression, defined as a persistent feeling of sadness, is a leading cause of disability worldwide (1). Studying social determinant risk factors for depression can help identify effective interventions and reduce disease burden; education, in particular, offers many opportunities for intervention (2). Lower educational attainment is associated with increased risk of depressive symptoms (3,4). For monozygotic twins, having a college degree was associated with fewer depressive symptoms, suggesting this association may persist independent of other social and genetic factors (5).

Education may affect depression through various socioeconomic pathways. First, people with less schooling may have fewer economic and social resources to address depressive episodes (6). Second, education affects socioeconomic position and people who ranked themselves lower in the social milieu, based in part on educational attainment, had higher odds of depression than those with higher social ranks (7). Third, education increases access to employment opportunities that are more creative, mentally stimulating, and involve higher autonomy, which also may affect mental well-being (8). These and other benefits of education for health and well-being can accumulate over one’s life (8).

Our study sought to address some remaining key gaps in research on education and depression. We proposed a theoretical framework, weaving together the research literature with our hypotheses (Figure). First, existing research often omits key factors in early childhood and adolescence that could confound the association between education and depression, including parents’ education, geographic location, and immigration (9,10). Additionally, socioeconomic factors during adulthood (eg, income, wealth, family size, marital status) likely mediate this association (8). Researchers must account for these factors to better understand the direct effect of education on depression and the indirect effects arising from adult socioeconomic position.

**Figure:**
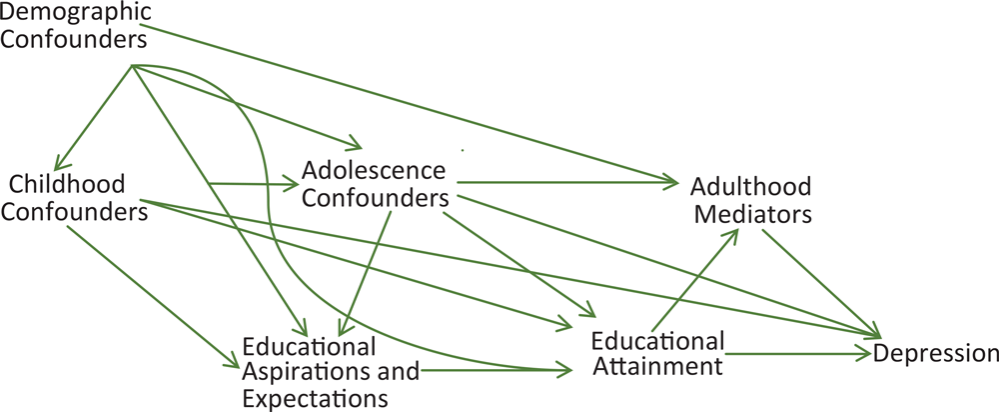
Theoretical framework consisting of hypothesized relationships between educational attainment, educational aspirations and expectations, depression in adulthood, and potential confounding and mediating variables.

Second, many studies do not assess potential effect measure modification. The theory of resource substitution posits that education is protective against disease for people with disadvantaged backgrounds (11). An inverse association between educational attainment and depression may be stronger in women than men, but few studies have assessed this association (12). Additionally, racial/ethnic variations are poorly understood. Some evidence suggests that education is inversely associated with depression for both white and black Americans (10). In another study, race/ethnicity and sex modified the association between educational attainment and mental health (13). Given this paucity of evidence, we investigated whether or not education–depression associations varied by sex or by race/ethnicity.

Third, most existing research fails to tease apart the dimensions of education that are dictated by individual determination versus external forces beyond individual control (14). These 2 different dimensions of education, operationalized as educational aspirations (individual determination) and educational expectations (external forces beyond one’s control) could affect health differently (15). Educational aspirations, or the level of education a person wants to attain, reflect a mixture of traits (eg, personal motivation, self-esteem) that may propel a person to educational achievement. Educational expectations, the level of education a person anticipates attaining, represent underlying structural factors that influence achievement (eg, childhood socioeconomic position, parental support) (14). Adolescents with increased educational expectations have more capacity for seeking out economic and social resources essential to better physical and mental health (16), whereas those with unfulfilled expectations may have increased depressive symptoms (17) (although this may be fully explained by lower educational attainment [18]). However, little is known about how adolescent educational aspirations and expectations are associated with health outcomes during adulthood, particularly mental health.

The National Longitudinal Survey of Youth 1979 (NLSY79) Cohort (19) provides an opportunity to test 3 related hypotheses. First, previous studies of the association between educational attainment and depression may not have adjusted for all important social factors that were confounders. We hypothesized that educational attainment will be inversely associated with depression and that this association will be attenuated when we adjust for confounders and mediators from across the life course. Second, the association between educational attainment and depression is independent of any relationship between depression and educational aspirations and expectations. We hypothesized that the association between educational attainment and depression would be attenuated and possibly disappear after adjusting for educational aspirations and expectations. Third, past studies suggest that sex and race/ethnicity may modify the effect of the adjusted association between educational attainment and depression. We hypothesized that the association between educational attainment and depression may be stronger among women and may vary by race/ethnicity.

## Methods

We used NLSY79 data, a cohort of people followed by the US Bureau of Labor Statistics and weighted to be nationally representative of Americans aged 14 to 21 in 1979. Participants in this age group were followed beginning in 1979 and completed surveys every 1 to 2 years thereafter. We used data through 2008; more sample details exist elsewhere (19). Briefly, we used a multistage stratified probability sampling approach to create a representative sample of noninstitutionalized civilians aged 14 to 21 in 1979 and to oversample youth from underrepresented groups (Hispanic/Latino, black, economically disadvantaged whites). NLSY79 also developed weights to allow for nationally representative estimates (19). We restricted our sample to include youth with complete data for our variables of interest, leaving a final sample of 4,417. This sample consisted of 56.9% of NLSY79 participants who were followed through 2008 and 44.3% of the original sample, which is comparable to other longitudinal studies over a similar timespan (14). The study was deemed exempt by the University of California Berkeley Committee for the Protection of Human Subjects, because the data are publicly available and nonidentifiable.

Our outcome of interest was depressive symptoms at age 40, as measured by the Center for Epidemiologic Studies Depression Scale Short Form, a 7-item scale measuring frequency of depressive symptoms in the prior week (<1, 1–2, 3–4, or 5–7 days/week). The 7-item scale is similarly reliable and precise as the original longer version, with improved internal consistency (20). The scores of these 7 items were summed; the total score could range from 0 to 21. If any item was missing, the total score was coded as missing. A cutoff total score of ≥8 was used to create a dichotomous variable from the scale (20).

Explanatory variables were educational attainment, aspirations, and expectations. Educational attainment was the number of years of education attained by age 25; the US Census assumes people complete education by age 25. Number of years of education attained was reported at each interview. Educational aspirations and expectations were assessed in 1979 as the number of years of education participants aged 14 to 21 aspired to or expected to attain. The 3 educational variables were categorical, classified as having or aspiring to or expecting to attain less than a high school education (<12 years of school), high school graduation (12–15 years of school), or college graduation (≥16 years of school). Maternal and paternal educational attainment and the highest parental education variables were coded in the same categorical manner.

Models adjusted for sex and race/ethnicity (black, Hispanic, and non-Hispanic white). Asians (n = 142) were excluded from the sample because of the small sample size and missing data. Childhood and adolescent confounders were maternal and paternal education, highest education level of either parent, speaking a foreign language as a child, being born outside of the United States, living in the South (Alabama, Arkansas, Delaware, District of Columbia, Florida, Georgia, Kentucky, Louisiana, Maryland, Mississippi, North Carolina, Oklahoma, South Carolina, Tennessee, Texas, Virginia, or West Virginia), and living in an urban area as a child. Each of these variables could be associated with access to educational opportunities. Participant age in 1979 was included to account for any potential birth cohort differences. We also tested for effect measure modification by sex and race/ethnicity.

Potential mediators from adulthood were included to estimate direct versus indirect effects of education on depression. Individual and total family wealth (wealth = assets – debts) and income were measured continuously in standardized year-2000 dollar increments and were log-transformed. We also included family size, number of dependents, marital status, living in the South as an adult, and living in an urban area as an adult, all as measured at age 40.

We used generalized linear modeling with the log linear link function to calculate adjusted risk ratios (RRs) (21) in Stata 14.1 (StataCorp). We specified a Poisson distribution with robust standard errors, which does not require accurately specifying the distribution to calculate accurate point estimates and standard errors and avoids nonconvergence issues (22). *P* values were 2-sided and unadjusted for multiple comparisons (23). We did sensitivity analyses by re-running all our models in a multiply imputed dataset by using Stata’s multiple imputation package. Five imputations of all education variables and confounders were conducted; our outcome, depression, was not imputed.

## Results

Approximately 16.2% of the 4,417 study participants had depression at age 40 (Table 1). By age 25, 66.5% were high school graduates, 24.2% graduated from college, and 9.3% did not complete high school. Among those excluded from the analyses, there was a similar proportion of high school graduates (66.1%), fewer college graduates (17.4%), more with less than a high school education (16.4%), and a higher prevalence of depression (20.8%) than among those included in the sample. The sample was evenly divided by sex (51.5% female) and most of the sample (84.9%) was white; for those excluded from the sample, a smaller proportion were women and a larger proportion were nonwhite. Compared with participants categorized as not depressed, the depressed group in the sample had a higher proportion of women, had parents who were less educated, were less educated themselves, had lower educational aspirations and expectations, and had less wealth, income, and total family income. Fewer of the depressed group were married.

**Table 1:** Weighted Descriptive Statistics for Complete Case Analysis Sample (N = 4,417) by Depression Status at Age 40, National Longitudinal Survey of Youth 1979 Cohort^a^, United States, 1979–2008

Weighted Proportion of Sample	Total, N = 4,417 (100%)	Not Depressed, n = 3,590 (83.8%)	Depressed, n = 827 (16.2%)	*P* Value^b^	Excluded From Analytic Sample (No. Range, 2,117–8,269)^c^
**Demographic Characteristics**					
**Age in 1979, y, mean (SD)**	17.6 (2.32)	17.6 (2.27)	17.7 (2.54)	.58	17.9 (2.33)
**Female, %**	51.5	49.3	63.3	< .005	47.2
**Race/ethnicity, %**					
Non-Hispanic white	84.9	86.1	78.9	< .005	75.9
Black/African-American	10.8	9.9	15.3		16.0
Asian	0	0	0		2.0
Hispanic/Latino	4.3	4.0	5.8		6.1
**Early Life Characteristics**					
**Father’s education, %**					
Less than high school graduate	31.7	29.8	41.0	< .005	34.7
High school graduate	49.1	50.3	42.5		47.0
College graduate	19.3	19.8	16.6		18.3
**Mother’s education, %**					
Less than high school graduate	28.5	26.4	39.4	< .005	35.0
High school graduate	60.3	62.2	50.3		56.1
College graduate	11.2	11.4	10.4		9.0
**Highest education of either parent, %**					
Less than high school graduate	19.1	17.4	27.7	< .005	27.5
High school graduate	58.3	59.5	52.2		66.7
College graduate	22.6	23.1	20.1		5.8
**Spoke a foreign language as child, %**	12.1	11.7	14.3	.06	16.1
**Born outside the United States, %**	3.5	3.4	4.0	.47	5.6
**Lived in the South^d^ as a child, %**	29.7	29.1	29.3	.41	33.0
**Lived in an urban (city or town) setting as a child, %**	77.1	77.0	77.9	.65	78.4
**Adolescent Characteristics**					
Educational aspiration (mean, SD)	14.6 (2.18)	14.7 (2.13)	14.3 (2.39)	< .005	14.4 (2.31)
Educational expectation (mean, SD)	14.1 (2.25)	14.2 (2.20)	13.5 (2.40)	< .005	13.8 (2.42)
Lived in an urban area (city or town) as an adolescent, %	77.8	77.9	76.8	.56	79.4
**Region of residence in 1979, %**					
Northeast	20.0	20.1	19.0	.49	22.6
North Central	34.3	34.7	32.1		26.2
South	29.7	29.3	31.6		34.2
West	16.1	15.9	17.2		17.0
**Adult Characteristics**					
**Educational attainment, %**					
Did not graduate from high school by age 25	9.3	7.9	16.9	< .005	16.4
Graduated from high school by age 25	66.5	66.1	68.1		66.1
Graduated from college by age 25	24.2	26.0	15.0		17.4
**Income status, mean (SD)**					
Wealth^e^ at age 40, $	223,721.90 (428,327.20)	223,050.60 (439,821.70)	120,332.00 (319,637.00)	< .005	145,253.40 (316,659.20)
Natural log of wealth at age 40	10.4 (3.6)	10.7 (3.2)	8.9 (4.7)	< .005	9.1 (4.4)
Annual income at age 40, $	43,849.38 (36,587.02)	45,895.20 (36,835.58)	33,289.73 (32,157.94)	< .005	32,619.15 (36,341.67)
Natural log of income at age 40	10.1 (2.0)	10.2 (1.9)	9.6 (2.6)	< .005	8.6 (3.7)
Total family annual income at age 40, $	70,776.56 (65,277.95)	74,428.33 (66,033.19)	51,927.63 (55,130.89)	< .005	57,435.72 (57,686.93)
Natural log of total family income at age 40	10.7 (1.7)	10.8 (1.5)	10.1 (2.3)	< .005	10.2 (2.2)
**Family household size at age 40, mean (SD)**	3.3 (1.5)	3.3 (1.5)	2.9 (1.6)	< .005	3.2 (1.6)
**Number of dependents at age 40, mean (SD)**	1.5 (1.3)	1.5 (1.2)	1.3 (1.3)	< .005	1.3 (1.3)
**Married at age 40, %**	67.4	70.1	53.4	< .005	32.0
**Lived in an urban area at age 40, %**	68.1	68.1	68.1	1.00	68.8
**Lived in the South^d ^at age 40, %**	34.0	33.6	36.1	.24	40.2

Abbreviation: SD, standard deviation.

a National Longitudinal Survey of Youth 1979 Cohort (19).

b Calculated by *t* test or χ^2 ^test.

c Range in sample size for the percentages calculated here, from the smallest amount of missingness for a variable, 2,117, to the largest, 8,269.

d Alabama, Arkansas, Delaware, District of Columbia, Florida, Georgia, Kentucky, Louisiana, Maryland, Mississippi, North Carolina, Oklahoma, South Carolina, Tennessee, Texas, Virginia, or West Virginia.

e Wealth = assets – debts.

We found no effect measure modification by race/ethnicity (Wald test *P* value = .50) or sex (Wald test *P* value = .88) for associations between levels of educational attainment at age 25 and depression at age 40 (Table 2). In our unadjusted model, higher educational attainment was associated with a lower risk of depression. The point estimate did not change substantially when we added child and adolescent confounders, although the childhood covariates still meaningfully contributed to the model (Wald *P* value < .005). Adding the hypothesized mediators attenuated the risk ratios. College graduates and high school graduates still had a lower risk of depression (adjusted RR = 0.73; 95% CI, 0.56–0.96) than those with less than a high school diploma (adjusted RR, 0.75; 95% CI, 0.62–0.91).

**Table 2:** Depression at Age 40 by Educational Attainment, Aspirations, and Expectations at Age 25, National Longitudinal Survey of Youth 1979 Cohort^a^

Variable	Risk Ratio (95% CI)	*P* Value
**Educational attainment, unadjusted**		
College graduate versus high school graduate	0.61 (0.48–0.77)	<.001
College graduate versus less than high school graduate	0.34 (0.26–0.45)	
High school graduate versus less than high school graduate	0.57 (0.47–0.68)	
**Educational attainment adjusted for sex only, Wald test value for sex, *P *< .005**		
College graduate versus high school graduate	0.62 (0.49–0.78)	<.001
College graduate versus less than high school graduate	0.34 (0.26–0.45)	
High school graduate versus less than high school graduate	0.56 (0.46–0.67)	
**Educational attainment adjusted for child covariates^b^; Wald test value for child covariates, *P *< .005**		
College graduate versus high school graduate	0.62 (0.48–0.81)	<.001
College graduate versus less than high school graduate	0.39 (0.28–0.52)	
High school graduate versus less than high school graduate	0.62 (0.51–0.75)	
**Educational attainment adjusted for child and adolescent covariates^c^; Wald test value for adolescent covariates, *P *< .56**		
College graduate versus high school graduate	0.62 (0.48–0.81)	<.001
College graduate versus less than high school graduate	0.39 (0.29–0.53)	
High school graduate versus less than high school graduate	0.62 (0.51–0.75)	
**Educational attainment adjusted for child, adolescent, and adult covariates^d^; Wald test value for adult covariates, *P* < .005**		
College graduate versus high school graduate	0.73 (0.56–0.96)	<.05
College graduate versus less than high school graduate	0.55 (0.40–0.75)	<.001
High school graduate versus less than high school graduate	0.75 (0.62–0.91)	<.01
**Educational aspirations adjusted for child, adolescent, and adult covariates**		
College graduate versus high school graduate	0.93 (0.79–1.10)	>.05
College graduate versus less than high school graduate	0.71 (0.42–1.19)	
High school graduate versus less than high school graduate	0.76 (0.46–1.27)	
**Educational expectations adjusted for child, adolescent, and adult covariates**		
College graduate versus high school graduate	0.82 (0.68–0.98)	<.05
College graduate versus less than high school graduate	0.59 (0.44–0.79)	.001
High school graduate versus less than high school graduate	0.72 (0.56–0.93)	<.05
**Educational attainment adjusted for child, adolescent, and adult covariates, plus educational aspirations and educational expectations**		
College graduate versus high school graduate	0.77 (0.58–1.02)	>.05
College graduate versus less than high school graduate	0.62 (0.44–0.88)	.01
High school graduate versus less than high school graduate	0.78 (0.58–1.04)	>.05

a National Longitudinal Survey of Youth 1979 (19). All adjusted models are adjusted for sex and race (except for sex-only models).

b Age in 1979, father’s educational attainment, mother’s educational attainment, highest educational attainment of either parent, speaking a foreign language as a child, being born outside of the United States, living in the South (Alabama, Arkansas, Delaware, District of Columbia, Florida, Georgia, Kentucky, Louisiana, Maryland, Mississippi, North Carolina, Oklahoma, South Carolina, Tennessee, Texas, Virginia, or West Virginia) as a child, and living in an urban setting as a child.

c Region of residence in the United States as an adolescent and living in an urban setting as an adolescent.

d Wealth (wealth = assets – debts) as an adult, income as an adult, total family income as an adult, family size as an adult, number of dependents as an adult, marital status as an adult, living in the South as an adult, and living in an urban setting as an adult.

We assessed associations between adolescent educational aspirations and expectations and depression. Although adolescent educational aspirations were not associated with adult depression, lower adolescent educational expectations were. People who expected to be college graduates had a lower risk of depression than those who expected to be high school graduates (adjusted RR = 0.82; 95% CI, 0.68–0.98) and those who expected to attain less than a high school diploma (adjusted RR = 0.59; 95% CI, 0.44–0.79). Those who expected to be high school graduates had a lower risk than those who did not expect to graduate from high school (adjusted RR = 0.72; 95% CI, 0.56–0.93). When we had educational attainment, aspirations, and expectations in the same model, those with a college degree had a lower risk of depression than those with less than a high school diploma (adjusted RR = 0.62; 95% CI, 0.44–0.88), but all of the other RRs between education variables and depression were no longer significant.

The analyses using the multiply imputed dataset (Table 3) largely confirmed findings of the complete case analyses (Table 2). All associations that were significant at the *P* = .05 level in the complete case analyses remained so in multiply imputed analyses. However, some additional associations emerged when using the multiply imputed dataset because of the larger sample size. In particular, significant associations between educational aspirations and depression emerged: those who aspired to a college level education were less likely to be depressed, compared with those who aspired to high school graduation or less than high school graduation. Additionally, in the models of educational attainment and depression where educational aspirations and expectations were included as covariates, all measures of association (ie, college graduation vs high school graduation, college graduation vs less than high school graduation, high school graduation vs less than high school graduation) were significant.

**Table 3:** Depression at Age 40, by Educational Attainment, Aspirations, and Expectations at Age 25 and Race/Ethnicity in the Multiply Imputed Dataset, National Longitudinal Survey of Youth 1979 Cohort^a^

Variable	Risk Ratio (95% CI)	*P* Value
**Educational attainment, unadjusted**		
College graduate versus high school graduate	0.59 (0.49–0.71)	<.001
College graduate versus less than high school graduate	0.34 (0.28–0.42)	
High school graduate versus less than high school graduate	0.58 (0.51–0.66)	
**Educational attainment adjusted for sex only; Wald test value for sex, *P* < .005**		
College graduate versus high school graduate	0.59 (0.49–0.71)	<.001
College graduate versus less than high school graduate	0.34 (0.27–0.41)	
High school graduate versus less than high school graduate	0.57 (0.50–0.65)	
**Educational attainment adjusted for child covariates^b^; Wald test value for child covariates, *P* < .005**		
College graduate versus high school graduate	0.61 (0.50–0.74)	<.001
College graduate versus less than high school graduate	0.37 (0.30–0.47)	
High school graduate versus less than high school graduate	0.61 (0.54–0.70)	
**Educational attainment adjusted for child^b^ and adolescent covariates^c^; Wald test value for adolescent covariates, *P* < .56**		
College graduate versus high school graduate	0.61 (0.50–0.75)	<.001
College graduate versus less than high school graduate	0.38 (0.30–0.47)	
High school graduate versus less than high school graduate	0.61 (0.54–0.70)	
**Educational attainment adjusted for child^b^, adolescent^c^, and adult covariates^d^; Wald test value for adult covariates, *P* < .005**		
College graduate versus high school graduate	0.70 (0.57–0.85)	<.001
College graduate versus less than high school graduate	0.51 (0.40–0.64)	
High school graduate versus less than high school graduate	0.72 (0.63–0.83)	
**Educational aspirations adjusted for child^b^, adolescent^c^, and adult^d^ covariates**		
College graduate versus high school graduate	0.83 (0.74–0.94)	>.05
College graduate versus less than high school graduate	0.63 (0.45–0.90)	
High school graduate versus less than high school graduate	0.76 (0.54–1.07)	
**Educational expectations adjusted for child^b^, adolescent^c^, and adult^d^ covariates**		
College graduate versus high school graduate	0.76 (0.66–0.87)	<.001
College graduate versus less than high school graduate	0.50 (0.41–0.62)	
High school graduate versus less than high school graduate	0.66 (0.56–0.79)	
**Educational attainment adjusted for child^b^, adolescent^c^, and adult^d^ covariates, plus educational aspirations and educational expectations**		
College graduate versus high school graduate	0.83 (0.71–0.97)	<.05
College graduate versus less than high school graduate	0.63 (0.48–0.82)	<.01
High school graduate versus less than high school graduate	0.76 (0.62–0.95)	<.05

a National Longitudinal Survey of Youth 1979 (19). All adjusted models are adjusted for sex and race (except for sex-only models).

b Child covariates are age in 1979, father’s educational attainment, mother’s educational attainment, highest educational attainment of either parent, speaking a foreign language as a child, being born outside of the United States, living in the South (Alabama, Arkansas, Delaware, District of Columbia, Florida, Georgia, Kentucky, Louisiana, Maryland, Mississippi, North Carolina, Oklahoma, South Carolina, Tennessee, Texas, Virginia, or West Virginia) as a child, and living in an urban setting as a child.

c Adolescent covariates are region of residence in the United States as an adolescent and living in an urban setting as an adolescent.

d Adult covariates are individual and total family wealth (wealth = assets – debts), income, family size, number of dependents, marital status, living in the South as an adult, and living in an urban area as an adult, all as measured at age 40.

## Discussion

After considering a comprehensive set of socioeconomic measures across the life course and exploring different dimensions of the educational experience in a nationally representative US longitudinal cohort, we concluded that higher educational attainment and educational expectations, but not educational aspirations, are associated with reduced risk of depression at age 40. Findings regarding educational expectations suggest that the social forces that constrain education may also affect health. In the NLSY79 cohort, the educational attainment–depression association did not vary by race/ethnicity or sex. Our findings add to the body of research on education and depression (24), and concur that “shooting for the stars” with educational expectations is not detrimental (18).

Adult socioeconomic position (ie, household income, wealth, family size, number of dependents) appears to partially mediate the association between educational attainment and depression, consistent with prior research on poverty and depression (24). Even after accounting for this mediation, an educational attainment–depression association remained, potentially via empowerment (25), social connections (26), stress (27), or a variety of other factors (eg, health behaviors, adult socioeconomic position, health literacy) (2,13).

Our study had limitations. First, these data were self-reported and observational and therefore susceptible to human error; we assume that any such error is nondifferential. Second, our primary analysis was a complete case analysis, which assumes that all missing data are missing completely at random. However, our sensitivity analyses using multiple imputation reached the same conclusions. Additionally, the excluded population was relatively similar to the study sample by a variety of childhood, adolescent, and adult characteristics. Our use of the NLSY79’s custom sampling weights also helped to counteract this limitation. Third, our generalizability is limited: almost 85% of participants in the NLSY79 sample were white. Although this was reflective of the population of adolescents in the United States in 1979, it is not reflective of the current US population. Fourth, we used the Baron and Kenny (28) approach to assess mediation, but other approaches to assess mediation also exist. Finally, we did not have a baseline measure of depression during adolescence, so we could not ascertain the incidence of depression. Therefore, we could only assess depression prevalence in adulthood, and could not know what depression began in adolescence or early adulthood, perhaps influencing educational attainment, expectations, or aspirations (ie, reverse causality or a more complex etiology). However, a systematic review of high school dropouts and mental health disorders suggested that depression is likely to be a result of low educational attainment rather than a cause of it (3).

Our study had several strengths. Our nationally representative, longitudinal cohort with detailed data across the life course, including multiple measures of socioeconomic position, allowed us to answer new and more nuanced questions related to the education–depression association. In particular, we had information about childhood socioeconomic position, which many other studies of adult mental health lack, and information about educational aspirations and expectations in adolescence, which are often not included in other education–health studies focused on educational attainment. Additionally, including adult mediating factors in our final model, particularly various measurements of adult socioeconomic position, allowed us to parse out the direct effects of education from the indirect effects mediated by the adult socioeconomic consequences of education. We also used adjusted risk ratios, not odds ratios, to report our findings, because risk ratios are more intuitive to interpret, more conservative, and more appropriate for non-rare outcomes (21).

Future researchers should continue to assess the nuances of education as a health determinant (29), including the use of even more granular categories of educational attainment. Additionally, as Americans increasingly are involved in education throughout their lifespan, even after age 25, future investigations could track educational trajectories and examine if a different age cut-off may be more appropriate for completion of educational attainment or if education attained after age 25 has similar or different health benefits. Additionally, given how the racial/ethnic makeup of the US population has changed since this cohort was begun, we encourage future studies to replicate these analyses in more diverse populations and populations of color to help inform developing interventions to reduce depression risks in the current US population.

Our set of analyses builds on similar work focused on obesity (14), with relatively similar findings. We encourage future researchers to continue to explore the potential roles (or lack thereof) of educational aspirations, expectations, and attainment in relation to other health outcomes in adulthood. Given the likely importance of historical and societal contexts for the social patterning we observed (30), we also encourage researchers to explore whether these associations persist for other generations in the United States and in other countries. Our study focused on adolescent educational aspirations and educational expectations, because these are most immediately relevant for educational attainment in late adolescence and young adulthood (when we assume the majority of educational attainment occurs), but future researchers could also assess whether changes in educational aspirations and educational expectations over the life course could further nuance our understanding of these phenomena.

Our findings support other research indicating that health and education practitioners should acknowledge educational interventions as public health interventions, and work together (2,30). Future research could experimentally assess such educational interventions to explore how increasing educational attainment may affect mental health.

Higher educational attainment and expectations, even after adjusting for potential confounders and mediators from across the lifespan, are associated with reduced risk of depression in mid-life in a nationally representative sample of US adults. We encourage future researchers to further explore the nuances of the educational experience as they relate to health outcomes over the lifespan, and we encourage practitioners to identify educational interventions that could have mental health benefits in subsequent decades.
